# Physicochemical properties and energy content of yellow dent corn from different climatic origins in growing pigs

**DOI:** 10.5713/ajas.19.0715

**Published:** 2019-12-24

**Authors:** Wenxuan Dong, Juntao Li, Zhongchao Li, Shuo Zhang, Xiaozhen Li, Chundi Yang, Ling Liu, Shuai Zhang

**Affiliations:** 1 State Key Laboratory of Animal Nutrition, China Agricultural University, Beijing 100193, China; 2 Yunnan Xiernan Feed Limited Liability Company, Kunming 650216, China

**Keywords:** Digestible Energy, Metabolizable Energy, Meteorological Condition, Growing Pigs, Yellow Dent Corn

## Abstract

**Objective:**

The objective of this study was to determine the digestible energy (DE) and metabolizable energy (ME) of yellow dent corn sourced from different meteorological origins fed to growing pigs and develop equations to predict the DE and ME of yellow dent corn from southwestern China.

**Methods:**

Sixty crossbred barrows were allotted to 20 treatments in a triplicate 20×2 incomplete Latin square design with 3 replicated pigs per dietary treatment during 2 consecutive periods. Each period lasted for 12 days, and total feces and urine during the last 5 days of each period were collected to calculate the energy contents.

**Results:**

On dry matter (DM) basis, the DE and ME in 20 corn grain samples ranged from 15.38 to 16.78 MJ/kg and from 14.93 to 16.16 MJ/kg, respectively. Selected best-fit prediction equations for DE and ME (MJ/kg DM basis) for yellow dent corn (n = 16) sourced from southwestern China were as follows: DE = 28.58–(0.12×% hemicellulose)+(0.35×% ether extract)–(0.83×MJ/kg gross energy)+(0.20×% crude protein)+(0.49×% ash); ME = 30.42–(0.11×% hemicellulose)+(0.31×% ether extract)–(0.81×MJ/kg gross energy).

**Conclusion:**

Our results indicated that the chemical compositions, but not the meteorological conditions or physical characteristics could explain the variation of energy contents in yellow dent corn sourced from southwestern China fed to growing pigs.

## INTRODUCTION

Corn is commonly used in commercial swine diets due to its high energy content and low concentrations of anti-nutritional factors compared with other cereal grains [1]. It is widely planted all around the world, with an annual production of over 1 billion tons worldwide [2]. Corns grown under drought-stressed conditions were reported to have greater contents of fibrous components compared with corns harvested under normal conditions [3]. In humid growing and harvest conditions, the nutritional values of corn could be affected by its mold contamination [4] and lower nutrient content [5]. The meteorological conditions under which the corn grows may have influence on the physicochemical properties of corn grains, thus affecting their nutrient properties used as feed ingredients for animals. Corn is one of the major energy sources supplied to pigs, which is directly related to the growth performance and carcass trait in commercial swine production [6]. It is reported that corn grains cultivated from various origins have different energy digestibility, energy retention rate and digestible energy (DE) and metabolizable energy (ME) values [7–10]. However, little information is available on the correlation between nutritional values and climatic origins of corn grains fed to growing pigs. Most previous studies focused on some special corn varieties and measured only 1 sample, thus, comprehensive evaluation of the DE and ME contents in yellow dent corn was limited [3–6,8–10].

We hypothesized that yellow dent corn grains grown under different climatic origins have different energy content and digestibility due to the environmental conditions, and that meteorological factors such as temperature, humidity and rainfall may be used as predictors to estimate the energy contents of corn fed to growing pigs. Thus, the objective of this study was to determine the energy contents of yellow dent corn produced from different climatic conditions, explore the relationships between energy contents and meteorological conditions and develop equations to predict the energy contents of yellow dent corns sourced from southwestern China.

## MATERIALS AND METHODS

All protocols used in this experiment were reviewed and approved by the Institutional Animal Care and Use Committee of China Agricultural University (Beijing, China). The animal trials were conducted at the Metabolism Laboratory of the Ministry of Agriculture Feed Industry Center (Chengde, China).

### Collection of corn samples

Sixteen yellow dent corn samples were collected from southwestern China (Yunnan, China) which account for 32% corn production in China and has diverse climatic conditions. Two yellow dent corn samples were obtained from northeastern China which account for 46% Chinese corn production and 2 yellow dent corn samples were obtained from Myanmar to provide as much variation as possible. Breeds and fertilization managements of corn samples collected in this study were kept as consistent as possible to eliminate systematic error.

The 20 corn grains were divided into 6 groups based on their geographical origins, including 2 tropical, 2 cold, 4 cool, 4 neutral, 4 warm and 4 hot areas. Detailed information about the origins of the corn samples used in this study is shown in Table 1.

### Animals, diets, and experimental design

The animal trial consisted 2 periods, in each period sixty crossbred growing barrows (Duroc×Large White×Landrace, with initial body weight of 29.7±3.5 kg for the first period and 34.9±5.6 kg for the second period) were allotted to 20 treatment diets in a triplicate 20×2 incomplete Latin square design with 3 replicated pigs per dietary treatment. Each period lasted for 12 days, including 7 days for experimental diet adaptation and 5 days for total urine and feces collection. Pigs received different experimental diets in each period. In addition, 7 days for metabolism crates adaptation and 5 days for diet transition using a fully balanced growing diet between each period were also included in the whole animal trial. All pigs were individually housed in stainless-steel metabolism crates (1.4×0.7×0.6 m) that were equipped with a nipple drinker and a feeder and placed in a temperature-controlled room with the temperature maintained at 22°C ±2°C. Daily feed allowance, equivalent to 4% of the body weight determined at the beginning of each period, was divided into 2 equal-sized meals provided at 0830 and 1530 hours, respectively. Water was provided *ad libitum* via the nipple drinker. The feed refusals and spillages were collected twice daily, dried, and weighed during the 5 days of urine and feces collection period.

The 20 treatment diets were formulated to contain 96.9% of one of the 20 corn samples as the only energy source (Table 2). The amount of vitamin and mineral premix was kept consistent in all diets and adjusted to meet the nutrient requirements of growing pigs recommended by NRC [11].

### Sample collection

Fecal samples were collected using plastic bags (one bag per pig) as soon as they appeared in the metabolism crates and stored at −20°C immediately. At the end of the collection period, the total 5 days fecal productions from each pig were blended and weighed. Approximately 300 g sub-samples of feces were weighed and dried in a forced-air drying oven at 65°C for 72 hours after thawing and mixing thoroughly. Sub-samples were stored at −20°C for further analysis after drying. Total urine was collected into plastic buckets (one bucket per pig) containing 50 mL of 6 mol/L hydrochloric acid (HCl) and placed under the metabolism crates. The HCl was added to reduce the nitrogen loss and limit the proliferation of bacteria. The volume of collected urine was measured every day and 10% of the daily urinary collection was stored at −20°C. Urine samples were pooled for each pig after the collection period. Approximately 45 mL sub-samples were collected for further chemical analysis. The fecal and feed samples were ground to pass through 40 mesh screens prior to proximate analysis and 60 mesh screens for AA analysis.

### Chemical analysis

Dry matter (DM; method 930.15), ash (method 942.15), crude protein (CP; method 990.03), ether extract (EE; method 920.39), crude fiber (CF; method 978.10), calcium (Ca; method 978.02) and phosphorus (P; method 946.06) of all corn samples were determined according to AOAC [12]. Filter bags (Model F57; Ankom Technology, Macedon, NY, USA) and fiber analyzer equipment (ANKOM200 Fiber Analyzer, Ankom Technology, USA) were used to determine the neutral detergent fiber (NDF) and acid detergent fiber (ADF) contents of the corn samples [13]. Hemicellulose was calculated as the difference between NDF and ADF concentrations. Total starch of all corn samples was analyzed using a commercial Starch Assay Kit (STA20; Sigma-Aldrich Corporation, St. Louis, MO, USA) according to method 7613.01 of the AOAC [12]. All corn samples were analyzed for AA according to the standard method described by AOAC [12]. Fifteen AAs were determined after hydrolysis with 6 mol/L HCl at 110°C for 24 hours using an AA Analyzer. Methionine and cysteine were determined as methionine sulphone and cysteic acid using an AA analyzer after cold performic acid oxidation overnight and hydrolyzing with 7.5 mol/L HCl at 110°C for 24 hours. Tryptophan was determined using high performance liquid chromatography (Agilent 1,200 Series, Santa Clara, CA, USA) after LiOH hydrolysis for 22 hours at 110°C. The corn grains were analyzed for bulk weight and 1,000 kernel weight according to Li et al [8]. The gross energy (GE) of feces, urine, diets, and corn samples were analyzed using an automatic isoperibol oxygen bomb calorimeter (model 6400; Parr1281 Calorimeter, Moline, IL, USA) calibrated by benzoic acid (26.45 MJ/kg). All analysis was conducted in duplicate.

### Calculations

The DE and ME contents of diets and corn grains as well as the apparent total tract digestibility (ATTD) of GE were calculated using the equations described by Adeola [14] as follows:

$$\begin{array}{l} \text{DEd }(\text{MJ}/\text{kg})=(\text{GEi}-\text{GEf})/\text{DMi};\\ \text{DEc }(\text{MJ}/\text{kg})=\text{DEd}/0.969;\\ \text{MEd }(\text{MJ}/\text{kg})=(\text{GEi}-\text{GEf}-\text{GEu})/\text{DMi};\\ \text{MEc }(\text{MJ}/\text{kg})=\text{MEd}/0.969;\\ \text{ATTD of GE }(\%)=(\text{GEi}-\text{GEf})/\text{GEi}, \end{array}$$

where DEd and MEd represent the DE and ME contents in each diet, respectively; GEi (MJ), GEf (MJ), and GEu (MJ) represent the total GE intake, GE output in feces, and GE output in urine, respectively; DMi represents dry matter intake (DMi); DEc and MEc represent the DE and ME contents in corn grains, respectively; 0.969 is the percentage of corn grains included in diets.

The net energy (NE) content of corn grains were calculated using the equation: NE (MJ/kg DM) = ([0.73×ME]+[1.33×EE]+[0.39×starch]–[0.62×CP]–[0.83×ADF])×0.0041868, in which chemical components are expressed as g/kg DM [15].

### Statistical analysis

Data were checked for normality using the UNIVARIATE procedure of SAS 9.2 (SAS Inst. Inc., Carry, NC, USA). No outliers were identified. Then data were analyzed using the PROC MIXED procedure of SAS. Dietary treatment was the only fixed effect and period was the random effect included in the model and the individual pig was treated as the experimental unit. There was no interaction between treatment and period. The LSMEANS statement was used to calculate the least squares means for each treatment with Tukey’s adjustment. The correlation coefficients (r) among the chemical compositions and energy values were calculated using the CORR procedure of SAS 9.2. Prediction equations for the DE and ME contents of corn grains were developed using the REG procedure of SAS 9.2. Stepwise regression was used with chemical compositions as independent variables. The R^2^, p-value, the Mallows statistic (C(p)), Bayesian information criterion (BIC), root mean square error (RMSE) and Akaike’s information criterion (AIC) were used to identify the best-fit equations. In all analyses, the differences were considered significant if p<0.05.

## RESULTS

### Nutrient composition

Variability of nutrient compositions among the 20 corn grains and among different geographical regions are shown in Table 3. On DM basis, the average CP, EE, CF, ash, NDF, ADF, starch, Ca, and P concentrations of 20 corn samples were 9.32%, 4.05%, 3.31%, 1.38%, 13.01%, 3.70%, 79.65%, 0.02%, and 0.27%, respectively. The average bulk weight and 1,000 kernel weight of corn grains were 757.2 g/L and 326.0 g on as-fed basis, respectively.

### Energy contents

The ATTD of GE in 20 corn grain samples ranged from 85.54% to 89.96% with a mean value of 87.65% (Table 4). On DM basis, the DE, ME and NE contents of the 20 corn grain samples averaged 16.16, 15.70, and 12.70 MJ/kg, respectively. The NE/ME and ME/DE ratios were consistent among corn grains and ranged from 0.80 to 0.81 and 0.97 to 0.98, respectively. In terms of corn sources, significantly greater (p<0.05) DE content was observed in source 10 compared with source 6. However, in terms of harvest regions, corn grains grown in the neutral area had greater (p<0.05) DE, ME, and NE contents than those grown in the area.

### Correlation coefficients and prediction equations

Correlation coefficients between chemical characteristics, physical characteristics, climatic coefficients, and energy content of corn grains are shown in Table 5. Positive correlation was detected between DE and ME contents (p<0.01, r = 0.86). In addition, EE was positively correlated with DE and ME contents (p<0.05, r = 0.56 and 0.46, respectively). The NDF showed negative correlation with DE and ME contents (p<0.05, r = −0.60, and −0.56, respectively). Hemicellulose was negatively correlated with DE and ME contents (p<0.01, r = −0.68 and −0.69, respectively). The GE also showed negative correlation with DE content (p<0.05, r = −0.46). Moreover, the environmental temperature of the cropping condition demonstrated negative correlation with the ash content in corn grains (p<0.05, r = −0.48). Otherwise, no significant correlation was observed between physicochemical or climatic conditions and energy contents in corn grains. The ATTD of GE was positively correlated with ME, DE, and ash content (p<0.05; r = 0.83, 0.90, and 0.49, respectively), but negatively correlated with the NDF and hemicellulose concentrations (p<0.01, r = −0.71, and r = −0.78, respectively).

The equations developed to predict the DE and ME contents and ATTD of GE in corn (n = 16) sourced from southwestern China are presented Table 6. The prediction equations with greater R2 and smaller RMSE, AIC, and BIC values were selected. The best-fit equations for DE, ME (MJ/kg DM), and ATTD of GE (%) in 20 corn samples evaluated in current study were: DE = 28.58–(0.12×hemicellulose)+(0.35×EE)− (0.83×gross energy)+(0.20×CP)+(0.49×ash), ME = 30.42–(0.11×hemicellulose)+(0.31×EE)–(0.81×gross energy), and ATTD of GE (%) = 122.83–(0.54×hemicellulose)+(0.66×EE) +(1.89×ash)–(1.92×gross energy), in which chemical components were expressed in % DM and GE were expressed as MJ/kg DM.

## DISCUSSION

### Nutrient composition

The physical property, chemical analysis, and AA concentrations in 20 corn grains observed in this study were within the range of values reported by previous studies [1,11,16–18].

In terms of physical characteristics, 1,000 kernel weight (coefficient of variance; CV = 9.18%) was more variable compared with bulk weight (CV = 3.10%), which agrees with the results reported by Li et al [9] and Newman et al [3]. Corn grain samples (i.e. sources 1 and 2) from Myanmar (tropical) had greater bulk weight and lower 1,000 kernel weight compared to corn grain samples obtained from China. Extreme temperatures or rainfall during the developmental stage of corn cobs can reduce the production and decrease the quality of the kernel [19–21]. However, we failed to draw a conclusion that physical characteristics of corn grains were affected by the cropping temperature or rainfall in the current study due to the lack of Burmese meteorological conditions. Similar results were also observed by Newman et al [3] that bulk weight and 1,000 kernel weight of corn grains were virtually identical between drought-stressed year and normal years.

The mean NDF and ADF contents observed in this study were greater than the values reported by NRC (10.3% and 3.3%, respectively), Sauvant et al (12.0% and 3.0%, respectively) and CVB (11.6% and 3.2%, respectively) [11,16,17]. But Bertol et al [22] reported that NDF concentrations of 2 lots was 15.09% and 13.57%, which were similar to the values observed in the current experiment, indicating that the nutrition compositions of corn grains grown nowadays may be different from those in the previous feedstuff tables. Newman et al [3] reported significantly lower ADF and NDF concentrations in drought-stressed corn grain compared with crops grown in normal conditions. However, Wang and Zhang [23] and Guo et al [24] concluded that there was no relationship between chemical compositions of corn and the meteorological factors. One possible explanation for these discrepancies may be the extreme weather conditions that have appeared in recent years all around the world, which may influence the development of corn cobs. As for the major energy-yielding composition, starch content of corn grains used in this study were also greater than the values in previous feedstuff tables. For example, the mean starch concentrations reported in NRC [11] and Sauvant et al [16] were 70.8% and 74.2% for yellow dent corn, respectively, and was 76.7% for NutriDense corn in NRC [11]. Otherwise, the values of other chemical constituents such as CP, EE, CF, Ash, Ca, and P were similar in our study compared with the previous reported values [11,16,17]. The development of corn breeding may also explain the change of starch and fiber contents in corn grown nowadays. The AA concentrations in corn samples used in the current study were similar to those values reported in previous studies [11,16,25,26]. For example, the average lysine content of corn samples used in the current study was 0.29%, which was consistent with the value of 0.28% in NRC [11] and Sauvant et al [16], and the CV of AA concentrations ranging from 6.52% to 16.35% were relatively low. Further exploration about the influence of meteorological conditions on AA concentrations in corn was not conducted due to the relatively consistent AA concentrations.

### Energy contents

No significant differences were observed in ATTD of GE among 20 different corn sources. However, in terms of harvest region, the ATTD of GE in corn grains harvested from neutral area were significantly greater than those from cool area. Park et al [10] evaluated the ATTD of GE in 9 corn grain sources and reported a range from 84.0% to 87.7%. Newman et al [3] demonstrated that the ATTD of GE in 28 drought-stressed corn grains varied from 80.6% to 85.6%. Both studies showed lower ATTD of GE compared with the results in the current study, which may result from the different corn breeds or experimental conditions. For some other cereal grains commonly used in swine diets, the ATTD of GE was reported to be greater than that of corn grains. For example, the ATTD of GE in 8 low-tannin sorghum cultivars ranged from 87.15% to 89.89% [27], and the ATTD of GE in 12 wheat cultivar ranged from 88.14% to 90.31% [28].

Those energy values in corn grains obtained from the current experiment were lower than the values in NRC [11] (16.35 MJ DE/kg, 16.09 MJ ME/kg, and 12.71 MJ NE/kg) and Sauvant et al [16] (16.42 MJ DE/kg, 16.08 MJ ME/kg, and 12.84 MJ NE/kg). Nevertheless, current results agreed with values in Park et al [10] which reported average 16.01 MJ DE/kg and 15.73 MJ ME/kg in 9 corn grain samples. In addition, different energy values in corn grains were reported in previous studies: 17.18 MJ DE/kg [29], 16.96 MJ DE/kg [9], 15.40 MJ DE/kg [3], 14.38 MJ DE/kg [22]; 16.42 MJ ME/kg [9], 15.15 MJ ME/kg [3]; 13.05 MJ NE/kg [18], 12.22 MJ NE/kg [3]. These differences in energy values among studies may be due to the various corn breeds and experimental conditions. Although differences in DE contents were observed among regions in this study, no difference was observed in ME or NE contents. This could be explained by the great deviation of the energy loss in urine. As a result, it was concluded that the available energy contents in corn grains are different among harvested regions and corn sources, which is consistent with previous studies [7,9,10].

### Correlation coefficients and prediction equations

It was reported that temperature and water stress had effects on the nutrient deposition in the cob formation in corn grains [21,24]. Different harvest environments could affect damaged to kernels [3]. It was also reported that physical characteristics were influenced by various meteorological conditions [23,24]. However, it is generally accepted that physical characteristics are not good parameters to predict the available energy contents in cereal grains fed to pigs compared with chemical characteristics [9,15,27]. It used to be widely believed that corn grown in northeastern China (Sources 3 to 4 in this study) had higher nutritional values compared with corn produced in southwestern China (Sources 5 to 20 in this study). The correlation analysis conducted in our study provided further proof that the geographical region is not directly related to the nutrient values of corn. We attribute the difference on energy content of corns from different regions to the chemical characteristics, animals, breeds and interactions between these factors [15].

As expected, EE was a positive predictor to estimate the energy contents in corn fed to growing pigs. Li et al [9] indicated that both EE and starch are reliable predictors in the estimation in DE and ME contents in corn. However, starch was not included in the prediction equations developed in the current study. Difficult to explain, similar results were also reported by Smith et al [29]. One possible explanation is that samples used herein was less variable on starch content compared with those in Li et al [9]. Moreover, GE was presented as a negative predictor for available energy estimations in the current equations, which was in consistent with equations reported by Pan et al [27], in which GE was also a negative predictor in estimating the energy contents in sorghum. Whereas both Li et al [9] and Zhao et al [28] reported a positive function of GE in predicting the available energy contents in corn and wheat, respectively. Above results indicated that GE was not as reliable as fiber components in energy contents prediction [15]. Previous studies suggested the superiority of NDF over ADF as a predictor in predicting DE and ME contents in corn fed to growing pigs [3,9,15], which is consistent with the current study. Hemicellulose, an important fiber fraction, showed strong correlation with DE and ME contents in corn. In addition, better goodness-of-fit (greater R^2^ and smaller RMSE) was observed when NDF was replaced by hemicellulose and other predictors were fixed in the prediction equations for DE, ME, and ATTD of GE. This observation concurs with findings on wheat [28], in which xylan was an important predictor in predicting DE and ME contents. Similarly, it has been suggested that over 60% of variation in DE content in 9 corn products could be explained by xylose, which is a portion of hemicellulose [30]. In addition, hemicellulose was also a reliable predictor in NE prediction [15].

Prediction equations could substantially reduce the need for animal trials in determining the DE and ME values of the feedstuffs fed to pigs, thus saving money and time [15,27]. Sixteen corn samples (i.e. sources from 5 to 20) originated from Yunnan Province, China were used to generate prediction equations to predict the DE and ME contents in corn from Yunnan Province. Thus, corn originated from other areas (i.e. sources from 1 to 4) were not adopted to generate prediction equations.

## CONCLUSION

This study indicated that the available energy contents in yellow dent corn originating from southwestern China were not correlated with the meteorological conditions such as temperature, humidity, or rainfall of the cropping environment. Chemical characteristics were more reliable factors compared with climatic origins or physical characteristics in predicting the DE and ME contents in yellow dent corn fed to growing pigs. This study also demonstrated the superiority of hemicellulose as a predictor in estimating the DE and ME values of corn grains.

## Figures and Tables

**Table 1:** Sources and associated climatic conditions of corn grains

Source	Geographic conditions			Meteorological conditions1)			Geographical division
	
	Region	Country	Longitude/Latitude	Temperature (°C)	Humidity (%)	Rainfall (mm)	
1	-	Myanmar	~20–22°N, 97–99°E2)	-	-	-	Tropical
2	-	Myanmar		-	-	-	Tropical
3	Changchun	China	43.90°N, 125.32°E	6.1	62	577.3	Cold
4	Jiamusi	China	46.82°N, 130.37°E	4.0	66	542.6	Cold
5	Xuanwei	China	26.22°N, 104.10°E	13.7	71	973.8	Cool
6	Huize	China	26.42°N, 103.30°E	13.0	70	791.3	Cool
7	Zhaotong	China	27.33°N, 103.72°E	11.8	75	674.7	Cool
8	Dongchuan	China	26.08°N, 103.18°E	12.8	58	741.3	Cool
9	Yongsheng	China	26.68°N, 100.75°E	13.7	68	950.8	Neutral
10	Yulong	China	26.82°N, 100.23°E	12.9	62	980.2	Neutral
11	Deqin	China	28.48°N, 98.92°E	6.3	68	650.8	Neutral
12	Weixi	China	27.18°N, 99.28°E	11.8	69	971.5	Neutral
13	Yuxi	China	24.35°N, 102.55°E	16.3	73	908.9	Warm
14	Qujing	China	25.50°N, 103.80°E	15.1	69	944.6	Warm
15	Chuxiong	China	25.03°N, 101.55°E	-	-	-	Warm
16	Dali	China	25.60°N, 100.23°E	15.1	68	1,054.8	Warm
17	Wenshan	China	23.37°N, 104.25°E	18.4	75	974.7	Hot
18	Lincang	China	23.88°N, 100.08°E	17.8	70	1,148.9	Hot
19	Puer	China	23.07°N, 101.03°E	-	-	-	Hot
20	Honghe	China	23.37°N, 103.40°E	20.6	74	832.2	Hot

1) All data were retrieved from http://data.cma.cn/ (Access time: Oct. 1, 2018; time period of observation: from 2008 to 2018).

2) Approximate longitude and latitude of the major corn production area in Myanmar.

**Table 2:** Ingredient compositions of the experimental diets (% as-fed basis)

Ingredient	Levels
Corn	96.90
Dicalcium phosphate	1.70
Limestone	0.60
Salt	0.30
Vitamin and mineral premix1)	0.50

1) Premix provided the following quantities per kilogram of complete diet: vitamin A as retinyl acetate, 5,512 IU; vitamin D_3_ as cholecalciferol, 2,200 IU; vitamin E as DL-alpha-tocopheryl acetate, 30 IU; vitamin K_3_ as menadione nicotinamide bisulfite, 2.2 mg; vitamin B_12_, 27.6 μg; riboflavin, 4 mg; pantothenic acid as DL-calcium pantothenate, 14 mg; niacin, 30 mg; choline chloride, 400 mg; folacin, 0.7 mg; thiamin as thiamine mononitrate, 1.5 mg; pyridoxine as pyridoxine hydrochloride, 3 mg; biotin, 44 μg; Mn as MnO, 40 mg; Fe as FeSO_4_·H_2_O, 75 mg; Zn as ZnO, 75 mg; Cu as CuSO_4_·5H_2_O, 100 mg; I as KI, 0.3 mg; Se as Na_2_SeO_3_, 0.3 mg.

**Table 3:** Variability of nutrient compositions among 20 corn grain samples with different climatic origins

Items	Mean	Min	Max	SD	CV	Means of geographical divisions1)					
						Tropical	Cold	Cool	Neutral	Warm	Hot
DM	88.21	86.78	89.65	0.62	0.71	87.68	88.93	87.88	88.33	88.44	87.98
GE (MJ/kg DM)	18.40	18.09	18.68	0.13	0.73	18.48	18.29	18.52	18.36	18.32	18.41
Chemical composition (% DM)											
CP	9.32	8.34	10.28	0.52	5.55	9.32	8.79	9.08	9.72	9.58	9.15
EE	4.05	2.81	4.98	0.56	14.01	4.48	3.89	4.16	4.63	3.52	3.76
CF	3.31	2.67	3.71	0.26	7.87	3.49	2.85	3.46	3.33	3.40	3.17
Ash	1.38	1.09	1.96	0.18	13.04	1.49	1.69	1.28	1.45	1.27	1.34
NDF	13.01	10.18	14.86	1.30	10.01	13.40	11.21	13.66	12.34	13.16	13.57
ADF	3.70	2.04	4.58	0.46	12.55	3.85	2.84	3.81	3.83	3.95	3.58
HC	9.30	10.75	7.20	1.08	11.65	9.55	8.37	9.37	8.51	9.21	9.99
Starch	79.65	75.09	82.65	1.93	2.42	76.29	80.30	80.32	78.41	80.77	80.46
Ca	0.02	0.01	0.03	<0.01	35.78	0.02	0.01	0.01	0.02	0.02	0.02
P	0.27	0.20	0.33	0.03	13.77	0.31	0.30	0.27	0.26	0.28	0.23
Physical characteristic, as-fed basis											
Bulk weight (g/L)	757.2	706.5	799.5	23.43	3.10	798.0	721.5	745.1	753.9	763.1	757.8
TKW (g)	326.0	265.0	377.5	29.94	9.18	265.0	307.5	342.5	338.1	313.8	337.5
Indispensable amino acid (% DM)											
Arginine	0.40	0.24	0.51	0.07	16.38	0.35	0.38	0.46	0.44	0.41	0.35
Histidine	0.28	0.23	0.38	0.04	14.47	0.29	0.24	0.27	0.29	0.26	0.33
Isoleucine	0.33	0.28	0.41	0.03	9.07	0.32	0.30	0.32	0.36	0.33	0.31
Leucine	1.12	0.93	1.45	0.12	11.07	1.11	1.02	1.12	1.25	1.12	1.03
Lysine	0.29	0.25	0.37	0.03	9.79	0.29	0.27	0.27	0.33	0.29	0.31
Methionine	0.20	0.16	0.25	0.02	11.21	0.21	0.18	0.20	0.19	0.21	0.21
Phenylalanine	0.43	0.36	0.56	0.04	9.30	0.42	0.38	0.44	0.47	0.44	0.41
Threoine	0.33	0.28	0.42	0.03	8.85	0.33	0.30	0.33	0.37	0.33	0.32
Tryptophan	0.07	0.06	0.10	0.01	16.35	0.06	0.07	0.08	0.07	0.07	0.07
Valine	0.44	0.37	0.52	0.03	7.23	0.44	0.40	0.44	0.48	0.44	0.43
Dispensable amino acid (% DM)											
Alanine	0.56	0.48	0.71	0.05	9.12	0.55	0.52	0.55	0.62	0.56	0.53
Aspartic acid	0.57	0.52	0.69	0.04	7.06	0.55	0.56	0.56	0.61	0.57	0.55
Cysteine	0.20	0.16	0.24	0.02	9.47	0.19	0.17	0.20	0.20	0.21	0.22
Glutamic acid	1.52	1.28	1.93	0.15	9.68	1.51	1.40	1.53	1.67	1.53	1.43
Glycine	0.29	0.24	0.32	0.02	6.52	0.29	0.25	0.29	0.29	0.29	0.30
Proline	0.93	0.79	1.07	0.07	7.65	0.96	0.85	0.93	0.97	0.91	0.97
Serine	0.40	0.34	0.50	0.04	10.19	0.40	0.37	0.39	0.44	0.40	0.38
Tyrosine	0.31	0.22	0.40	0.05	15.70	0.28	0.24	0.30	0.37	0.28	0.33

Min, minimum; Max, maximum; SD, standard deviation; CV, coefficient of variation; DM, dry matter; GE, digestible energy; CP, crude protein; EE, ether extract; CF, crude fiber; NDF, neutral detergent fiber; ADF, acid detergent fiber; HC, hemicellulose; Ca, Calcium; P, Phosphorus; TKW, thousand kernel weight.

1) Sources of corn grains are described according to its geographical divisions.

**Table 4:** Energy contents in the 20 corn grain samples with different climatic origins (dry matter basis)

Sources1)	DE (MJ/kg)	ME (MJ/kg)	NE (MJ/kg)	NE/ME	ME/DE	ATTD of GE (%)
Tropical						
1	16.26^ab^	15.65	12.62	0.81	0.98	87.06
2	16.03^ab^	15.67	12.73	0.81	0.97	87.67
Mean	16.30^xy^	15.84^xy^	12.76^xy^	0.81	0.97	87.75^xy^
Cold						
3	16.35^ab^	15.73	12.85	0.80	0.98	88.51
4	16.24^ab^	15.77	12.99	0.81	0.98	88.91
Mean	16.23^xy^	15.81^xy^	12.80^xy^	0.81	0.98	88.65^x^
Cool						
5	16.49^ab^	15.91	12.74	0.80	0.97	88.43
6	15.38^b^	14.93	12.82	0.81	0.98	85.73
7	16.02^ab^	15.72	13.03	0.81	0.98	86.81
8	15.87^ab^	15.52	12.53	0.81	0.97	86.82
Mean	15.93^y^	15.47^y^	12.54^y^	0.81	0.97	86.77^y^
Neutral						
9	16.68^ab^	16.01	12.16	0.81	0.97	88.99
10	16.78^a^	16.14	12.64	0.80	0.98	89.96
11	16.30^ab^	16.16	12.68	0.81	0.98	88.53
12	16.30^ab^	15.97	12.63	0.81	0.98	88.04
Mean	16.41^x^	15.97^x^	12.88^x^	0.81	0.98	88.75^x^
Warm						
13	16.49^ab^	15.90	13.09	0.81	0.97	88.43
14	15.88^ab^	15.66	12.88	0.81	0.98	87.25
15	15.94^ab^	15.60	12.72	0.81	0.98	87.47
16	16.13^ab^	15.44	12.85	0.81	0.97	87.48
Mean	16.12^xy^	15.67^xy^	12.63^xy^	0.81	0.97	87.72^xy^
Hot						
17	15.80^ab^	15.46	12.62	0.81	0.98	86.82
18	16.23^ab^	15.89	12.66	0.81	0.98	87.28
19	15.98^ab^	15.63	12.35	0.81	0.97	87.25
20	16.13^ab^	15.33	12.49	0.81	0.97	85.54
Mean	15.99^xy^	15.62^xy^	12.63^xy^	0.81	0.98	86.89^y^
SEM	0.02	0.03	0.02	<0.01	<0.01	0.28
p (source)	0.017	0.146	0.102	0.317	0.676	0.128
p (division)	0.011	0.032	0.049	0.518	0.394	0.002

DE, digestible energy; ME, metabolizable energy; NE, net energy; ATTD, apparent total tract digestibility; GE, gross energy; SEM, standard error of the mean.

1) Sources of corn grains are described according to its geographical divisions.

a,b Means among 20 corn grain sources differ without a common superscript (p<0.05).

x,y Means among 6 geographical divisions differ without a common superscript (p<0.05).

**Table 5:** Correlation coefficients among chemical characteristics, physical characteristics, climatic conditions and energy contents of 20 corn grain samples

Item	ME	DE	GE	Ash	CP	EE	NDF	ADF	HC	CF	Starch	BWT	TKW	Tem	Hum	Rainfall
DE	0.86**	-	-	-	-	-	-	-	-	-	-	-	-	-	-	-
GE	−0.41	−0.46*	-	-	-	-	-	-	-	-	-	-	-	-	-	-
Ash	0.44	0.40	−0.10	-	-	-	-	-	-	-	-	-	-	-	-	-
CP	0.19	0.26	−0.11	−0.13	-	-	-	-	-	-	-	-	-	-	-	-
EE	0.56*	0.46*	0.25	0.33	−0.12	-	-	-	-	-	-	-	-	-	-	-
NDF	−0.56*	−0.60**	0.45*	−0.17	−0.05	−0.10	-	-	-	-	-	-	-	-	-	-
ADF	−0.10	−0.19	0.32	−0.11	0.18	−0.02	0.61**	-	-	-	-	-	-	-	-	-
HC	−0.69**	−0.68**	0.47	−0.13	−0.08	−0.09	0.94	0.34	-	-	-	-	-	-	-	-
CF	−0.22	−0.18	0.55*	−0.21	0.31	0.20	0.44	0.58**	0.35	-	-	-	-	-	-	-
Starch	−0.35	−0.43	−0.06	−0.25	−0.17	−0.48*	0.18	0.02	0.16	−0.24	-	-	-	-	-	-
BWT	−0.14	−0.10	0.40	−0.39	0.11	0.09	0.10	0.21	0.12	0.34	−0.46*	-	-	-	-	-
TKW	−0.26	−0.25	0.38	−0.33	0.11	0.06	0.35	0.07	0.33	0.20	0.27	−0.29	-	-	-	-
Tem	−0.20	−0.25	−0.04	−0.48*	0.31	−0.31	0.35	0.26	0.31	0.16	0.45	−0.01	0.39	-	-	-
Hum	0.16	0.06	−0.02	−0.12	−0.03	−0.17	−0.31	0.01	−0.38	−0.38	0.19	−0.13	0.25	0.04	-	-
Rainfall	0.25	0.17	−0.04	−0.23	−0.18	−0.02	−0.18	0.14	−0.27	−0.31	0.08	0.01	0.36	0.26	0.90**	-
ATTD of GE	0.83**	0.90**	−0.48	0.49*	0.26	0.35	−0.71**	−0.17	−0.78**	−0.30	−0.26	−0.32	−0.22	−0.31	−0.37	0.20

ME, metabolizable energy; DE, digestible energy; GE, gross energy; CP, crude protein; EE, ether extract; NDF, neutral detergent fiber; ADF, acid detergent fiber; HC, hemicellulose; CF, crude fiber; BWT, bulk weight; TKW, thousand kernel weight; Tem, temperature; Hum, humidity; ATTD, apparent total tract digestibility.

* p<0.05;

** p<0.01.

**Table 6:** Prediction equations for digestible energy, metabolizable energy, and apparent total tract digestibility of gross energy in 20 corn grain samples with different climatic origins using stepwise regression (dry matter basis)

Item	Regression equations1)	Statistics					

		R^2^	C(p)	RMSE	AIC	BIC	p-value
Prediction equations for DE (MJ/kg)							
1	DE = 29.06–(0.09×NDF)+(0.37×EE)–(0.86×GE)+(0.21×CP)+(0.49×ash)	0.85	3.37	0.16	−57.38	−49.43	<0.01
2	DE = 28.58–(0.12×HC)+(0.35×EE)–(0.83×GE)+(0.20×CP)+(0.49×ash)	0.88	2.65	0.14	−60.85	−52.90	<0.01
Prediction equations for ME (MJ/kg)							
3	ME = 32.52–(0.07×NDF)+(0.33×EE)–(0.94×GE)	0.70	8.91	0.17	−66.53	−64.88	<0.01
4	ME = 30.42–(0.11×HC)+(0.31×EE)–(0.81×GE)	0.72	7.05	0.17	−56.65	−49.06	<0.01
Prediction equations for ATTD of GE (%)							
5	ATTD of GE = 132.12–(0.53×NDF)+(0.83×EE)+(1.81×ash)+(0.88×ADF)–(2.54×GE)	0.85	8.21	0.49	−23.89	−19.19	<0.01
6	ATTD of GE = 122.83–(0.54×HC)+(0.66×EE)+(1.89×ash)–(1.92×GE)	0.86	5.65	0.45	−22.74	−14.79	<0.01

DE, digestible energy; ME, metabolizable energy; ATTD, apparent total tract digestibility; GE, gross energy; C(p), the Mallows statistics; RMSE, root mean square error; AIC, Akaike’s information criterion; BIC, Bayesian information criterion; NDF, neutral detergent fiber; EE, ether extract; CP, crude protein; HC, hemicellulose; ADF, acid detergent fiber.

1) Values were determined with 6 observations per sample; chemical components were expressed as % dry matter; gross energy were expressed as MJ/kg dry matter.
